# Non-coding RNAs: The recently accentuated molecules in the regulation of cell autophagy for ovarian cancer pathogenesis and therapeutic response

**DOI:** 10.3389/fphar.2023.1162045

**Published:** 2023-03-31

**Authors:** Bi Peng, Jing Li, Yuanliang Yan, Yuanhong Liu, Qiuju Liang, Wei Liu, Abhimanyu Thakur, Kui Zhang, Zhijie Xu, Jian Wang, Fan Zhang

**Affiliations:** ^1^ Department of Pathology, Xiangya Hospital, Central South University, Changsha, Hunan, China; ^2^ Department of Pharmacy, Shanghai Pudong New Area People’s Hospital, Shanghai, China; ^3^ Department of Pharmacy, Xiangya Hospital, Central South University, Changsha, Hunan, China; ^4^ National Clinical Research Center for Geriatric Disorders, Xiangya Hospital, Central South University, Changsha, Hunan, China; ^5^ Department of Orthopedic Surgery, The Second Hospital University of South China, Hengyang, Hunan, China; ^6^ Ben May Department for Cancer Research, Pritzker School of Molecular Engineering, University of Chicago, Chicago, IL, United States; ^7^ State Key Laboratory of Silkworm Genome Biology, Medical Research Institute, Southwest University, Chongqing, China; ^8^ Department of Gynecology, Xiangya Hospital, Central South University, Changsha, Hunan, China

**Keywords:** non-coding RNAs, cell autophagy, ovarian cancer, cancer pathogenesis, prognosis

## Abstract

Autophagy is a self-recycling and conserved process, in which the senescent cytoplasmic components are degraded in cells and then recycled to maintain homeostatic balance. Emerging evidence has suggested the involvement of autophagy in oncogenesis and progression of various cancers, such as ovarian cancer (OC). Meanwhile, the non-coding RNAs (ncRNAs) frequently regulate the mRNA transcription and other functional signaling pathways in cell autophagy, displaying promising roles in human cancer pathogenesis and therapeutic response. This article mainly reviews the cutting-edge research advances about the interactions between ncRNAs and autophagy in OC. This review not only summarizes the underlying mechanisms of dynamic ncRNA-autophagy association in OC, but also discusses their prognostic implications and therapeutic biomarkers. The aim of this review was to provide a more in-depth knowledge framework exploring the ncRNA-autophagy crosstalk and highlight the promising treatment strategies for OC patients.

## Introduction

Ovarian cancer (OC) is a major threat to women’s health. With the deepening of research, the relationship between tumorigenesis and gene alterations has been gradually known and valued. It has been reported that nearly one-fifth of OC is mainly caused by BRCA1 and BRCA2 mutations (Norquist et al., 2016; Kuchenbaecker et al., 2017). Furthermore, OC is related to infertility, endometriosis, and the use of oral contraceptives and intrauterine devices (La Vecchia, 2017; Kralickova et al., 2020; Balayla et al., 2021). OC is characterized by strong concealment and high mortality (Kuroki and Guntupalli, 2020). It was estimated that there were more than 300,000 new OC cases and more than 200,000 deaths worldwide in 2020 (Cabasag et al., 2022). Most patients with OC have no symptoms in the early stage and are usually diagnosed with the metastatic and peritoneal spread in the late stage. In addition, the 5-year survival rate of OC patients is about 47% and has not improved in the past twenty years (Moufarrij et al., 2019). At present, the first-line treatment strategy for OC remains surgery, followed by chemotherapy using agents such as platinum and Taxus (Gogineni et al., 2021). Recently, bevacizumab and PARP inhibitors such as olaparib, rucaparib, and niraparib have received FDA approval for use in OC patients (O'Malley, 2019). Nevertheless, a high recurrence rate and chemotherapeutic resistance remain serious obstacles to treating OC (Norouzi-Barough et al., 2018). Hence, it is necessary to investigate the underlying mechanism of tumorigenesis and progression, as well as explore novel and effective therapeutic approaches. Recent studies have implied that non-coding RNAs (ncRNAs) play vital roles in the initiation and progression of OC through autophagy regulation.

The ncRNAs were originally regarded as “junk DNA” for a long time, possessing no functions (Sharma et al., 2022). However, with the breakthrough of sequencing technology, the roles of ncRNAs in humans were redefined (Costa, 2005; Chen et al., 2022a). Currently, it is well known that ncRNAs are unique molecules that could be used as protein transcription templates to generate functional biomarkers involved in physiological and pathological activities (Mattick and Makunin, 2006). They are mainly divided into three categories: microRNAs (miRNAs), long non-coding RNAs (lncRNAs), and circular RNAs (circRNAs). MiRNAs, with a length of about 21 nucleotides, have the functions of inhibiting transcription and degrading messenger RNAs (mRNAs) by interacting with the 3′-untranslated region of the target genes (Filipowicz et al., 2008). LncRNAs, which are greater than 200 nucleotides in length, participate in a wide variety of biological processes. Their functions mainly include acting as sponges and scaffolds and signaling (Stackhouse et al., 2020). CircRNAs are single-stranded, covalently closed molecules and can serve as competing endogenous RNAs (ceRNAs) or miRNA sponges to regulate biological activities (Zhou et al., 2020). LncRNAs and circRNAs contain binding sites for miRNAs and bind to them to inhibit their activity and functions (Salmena et al., 2011; Huang et al., 2018a).

Recent studies have suggested the functional roles of ncRNAs in the regulation of cell autophagy and participating in many kinds of cancers, including OC. For example, miR-145-3p could downregulate the expression level of HDAC4 to activate autophagic cell death in multiple myeloma (Wu et al., 2020). lncRNA GBCDRlnc1 can reduce the chemotherapy sensitivity of gallbladder cancer cells by activating cell autophagy (Cai et al., 2019). Overexpression of circATG7 has stimulative effects on cell autophagy in pancreatic cancer cells, thus promoting cancer progression (He et al., 2022). Meanwhile, in OC, lncRNA XIST could trigger cell autophagy by inhibiting the downstream target miR-506-3p, leading to enhanced resistance to carboplatin (Xia et al., 2022). In addition, the aberrant lnc-CTSLP8 displayed crucial roles in regulating cell autophagy and metastasis in OC cells (Wang et al., 2021a).

This paper reviews the effect of ncRNAs-associated signaling on the occurrence and development of OC by regulating autophagic processes. Furthermore, the underlying mechanisms and functional status of ncRNAs-autophagy interaction in prognosis and drug resistance of OC patients are also discussed. These findings would further improve our understanding of the ncRNA-autophagy axis in the malignant progression of OC and offer promising targets to enhance the therapeutic effect for OC.

### Functional roles of autophagy modulation

Autophagy is a highly conserved and complex process in all eukaryotes, which maintains the homeostasis of cells and organisms through degradation and recycling (Kim and Klionsky, 2000; Yu et al., 2018a; Peng et al., 2021). Autophagy is classified into three categories: micro-autophagy, macro-autophagy, and chaperone-mediated autophagy (CMA). In micro-autophagy, the lysosome membrane invaginates or protrudes to engulf cellular components directly (Mijaljica et al., 2011). Macro-autophagy refers to the isolation of cellular components from lysosomes. More specifically, double-membrane vesicles, namely autophagosomes, are newly synthesized to isolate cellular components and transport them to lysosomes (Yorimitsu and Klionsky, 2005). Dynamic membrane rearrangement is involved in both micro-autophagy and macro-autophagy (Wang and Klionsky, 2003). In addition, CMA uses molecular chaperones to recognize targeted proteins containing specific pentapeptide motifs (Massey et al., 2004; Yang et al., 2019). Three kinds of autophagy have their functions to maintain metabolism and internal balance together. Among the three types of autophagy, macro-autophagy (called ‘autophagy’ hereafter) is the most thoroughly studied and widely applied in human physiological and pathological processes.

**FIGURE 1:**
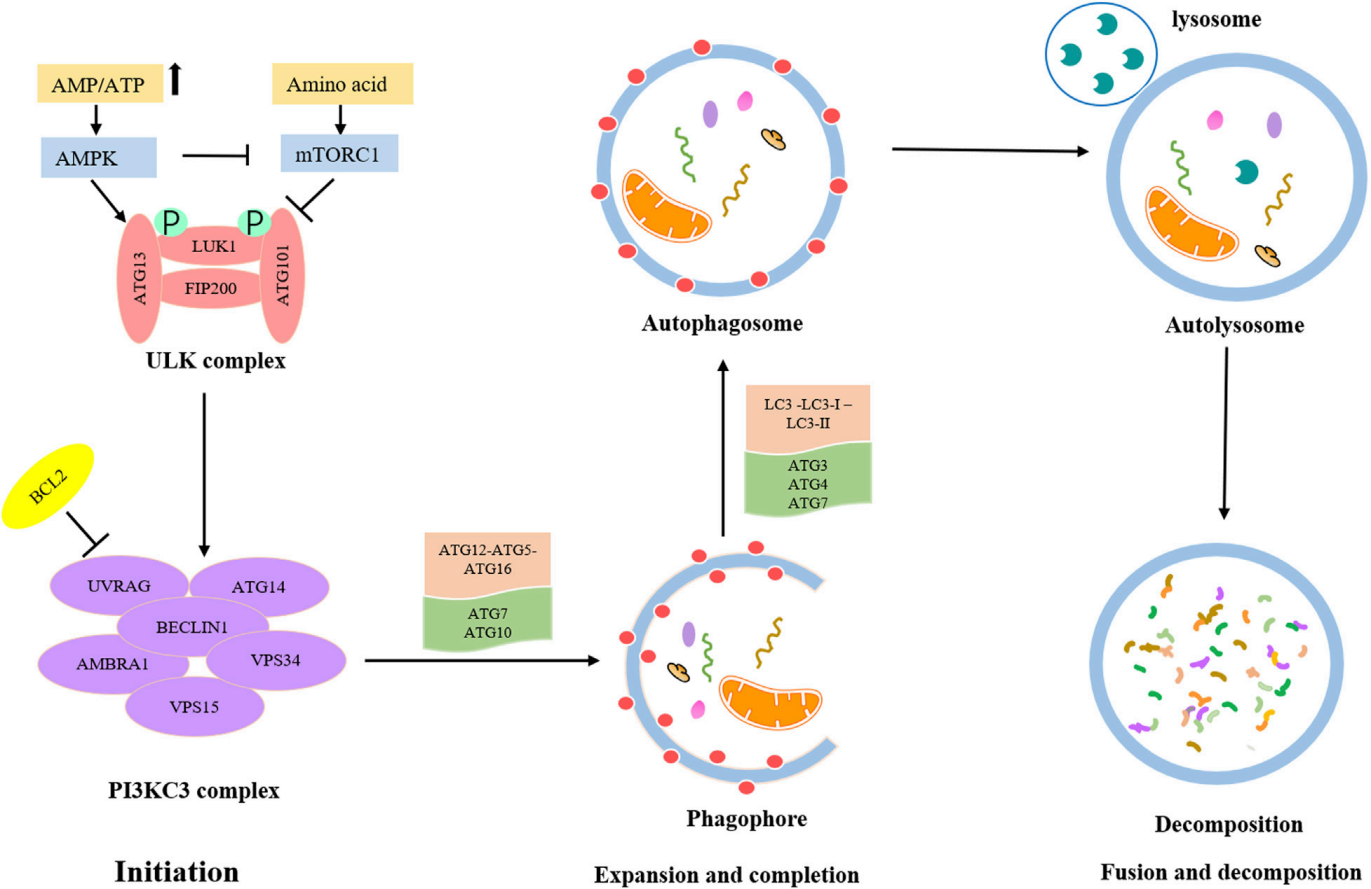
Overview of autophagy process.

The detailed process of autophagy is shown in Figure 1. Autophagy is initially triggered by various stress conditions, including hypoxia, oxidative stress, infection, nutritional deficiencies, and growth factor reduction. These autophagy-trigger signals cause the activation of MAPK and mTOR pathways, promoting or inhibiting the progression of autophagy. The process of autophagy can be divided into the following three main phases: (Norquist et al., 2016) Initiation phase, wherein the pre-initiation complex, unc-51-like kinase (ULK), composed of ULK1, ATG13, ATG101, and FIP200 is activated, which leads to the phosphorylation of the class III PI3K (PI3KC3) complex, consisting of BECN-1, UVRAG, AMBRA1, VPS15, and VPS34 (Russell et al., 2013). This complex recruits ATG9-containing vesicles to trigger phagophore nucleation. Meanwhile, the activated PI3KC3 complex can be inhibited by BCL2; (Kuchenbaecker et al., 2017) Expansion and completion phase, wherein the most critical step is to recognize cargo and lengthen phagophore, which is conducive to forming the autophagosome. At this stage, there are two vital ubiquitin–like conjugation pathways (Klionsky and Schulman, 2014). The first one involves the combination of ATG12 and ATG5 to form ATG5-ATG12 conjugate and interacts with ATG16L1, leading to the formation of the ATG12-ATG5-ATG16L1 complex. This ubiquitin–like conjugation pathway requires the participation of ATG7 and ATG10. The second stage consists of the cleavage of LC3 into LC3-Ⅰ with the help of ATG4, and then LC3-Ⅰ is converted into LC3-II with the catalysis of ATG7 and ATG3. Consequently, LC3-II is coupled with phosphatidylethanolamine to promote the expansion of the phagophore and the formation of autophagosomes; (La Vecchia, 2017) Fusion and decomposition phase, in which autophagosomes further fuse with lysosomes to form autolysosomes, where activated lysosomal hydrolase decomposes the substrate. Eventually, the degraded substrate is transferred to the cytoplasm for cell recycling.

Autophagy is a flexible mechanism of cell self-protection (Hill and Wang, 2022)**.** When human beings are in a state of stress, such as hunger, infection, and hypoxia, autophagy can be induced and provide energy for cellular activities (Parzych and Klionsky, 2014). Previous reports have summarized that autophagy dysfunction needs to be responsible for many diseases, such as neurodegenerative, cardiovascular, metabolic, and inflammatory disorders and cancers (Saha et al., 2018). Zhou et al. have proved that autophagy activation plays an important role in the pathogenic mechanism of lupus nephritis. It has a negative correlation with podocyte lesions, which is answerable to the progression of lupus nephritis (Zhou et al., 2019). Djajadikerta et al. have suggested that autophagy blockade is detrimental to the degradation of toxic protein aggregates involved in neurodegenerative diseases, such as amyotrophic lateral sclerosis, Alzheimer’s disease, and Parkinson’s disease. Autophagy activation may be beneficial in delaying the progression of these diseases by accelerating the breakdown of toxic protein aggregates (Djajadikerta et al., 2020). Accumulating evidence has also confirmed that autophagy is related to the occurrence, development, and treatment of various cancers. For example, autophagy can be used as an accelerator of monocarboxylate transporter 1 expression *via* the activation of the Wnt/β-catenin signaling pathway, promoting the metastasis of liver cancer (Fan et al., 2018). Autophagy can cooperate with TrkC/NT-3 axis to enhance the viability of hypoxic glioblastoma cells (Jawhari et al., 2017). The deletion of TTK protein kinase could inhibit cisplatin-induced autophagy *via* activating the mTOR signaling pathway, thus enhancing the sensitivity of OC cells to cisplatin (Qi et al., 2021). Collectively, autophagy plays a significant part in a wide variety of diseases. Targeting autophagy may provide a new strategy with wide application prospects for clinical management.

### ncRNA-autophagy signaling interaction in OC

The correlations between ncRNAs and cell autophagy in human cancers have been well recognized by numerous investigators (Liang et al., 2021). Elevated miR106A-5p is dedicated to the suppression of autophagy, which is correlated with late-stage, easy relapse, and undesirable prognosis of patients with nasopharyngeal carcinoma (Zhu et al., 2021). lncRNA EIF3J-DT can directly bind to ATG14 and reduce the degradation of ATG14. Thus, autophagy is activated, and chemotherapy resistance is enhanced in gastric cancer patients (Luo et al., 2021). circRNA CDYL is significant in facilitating cell proliferation of breast cancer by enhancing autophagy, leading to poor clinical outcomes (Liang et al., 2020). Additionally, extensive research has proved that ncRNAs also take part in the regulation of autophagy in many aspects and affect the progression of OC (Huang et al., 2018b). Vescarelli et al. have shown that overexpressed miR-200c enhances the sensitivity of OC cells to olaparib *via* regulating the expression of neuropilin 1 (Vescarelli et al., 2020). Song et al. have demonstrated that circRNF144B can strengthen Beclin-1 ubiquitination levels by modulating miR-342-3p and FBXL11, which results in the inhibition of autophagy and the promotion of OC progression (Song et al., 2022). Many researchers have speculated that ncRNAs have a great possibility of being developed as the targets for prognosis and treatment of OC. Therefore, to gain a deeper understanding, we concluded the possible mechanism and biological effects of ncRNAs associated with autophagy in OC, aiming to search for possible and promising therapeutic strategies and targets.

### Autophagy-associated miRNAs in ovarian cancers

**FIGURE 2:**
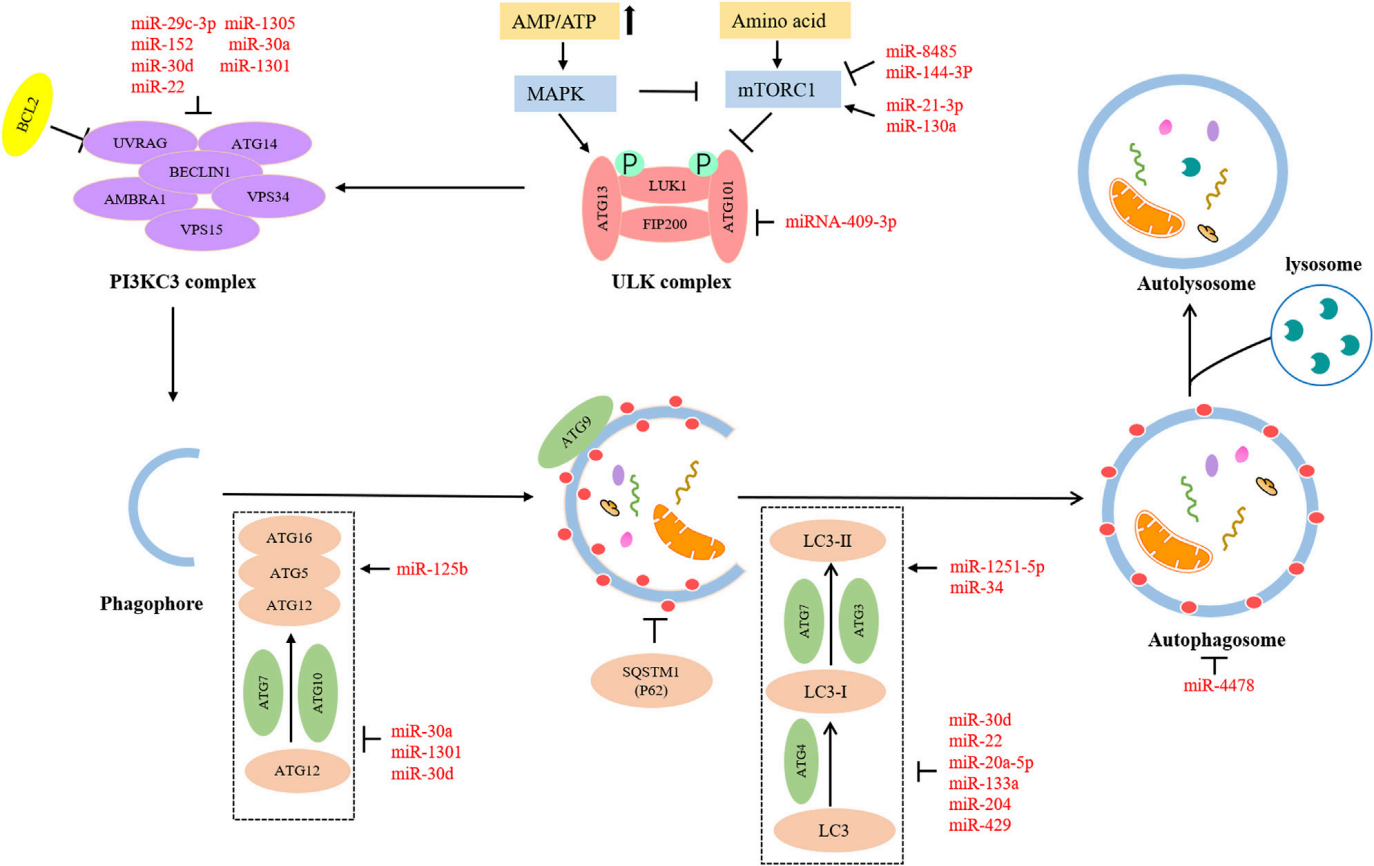
Roles of miRNAs in autophagy regulation in OC.

The miRNAs are an important participant in modulating gene expression, which affects cellular processes, and their aberrant expression contributes to the progression of cancers (Deb et al., 2018; Zhang et al., 2022a). Emerging evidence has indicated that aberrant miRNAs can affect the various stages of autophagy, thereby producing stimulative or suppressive effects on OC progression. Hence, we have summarized some miRNAs involved in autophagy in OC (Table 1; Figure 2).

**TABLE 1:** The miRNAs involved in autophagy modulation in OC.

miRNA	Possible targets	Autophagic effects	Effects on OC cells	Effects on OC samples	Models	Reference
miR-8485	LAMTOR3, mTOR, ATG13	Promotion	Facilitating apoptosis suppressing proliferation	—	Cell culture	Wang et al. (2022a)
miR-144-3p	IGF2R, AKT, mTOR	Promotion	—	—	Cell culture	Yuan et al. (2022)
					Animal model	
miR-130a	TSC1, mTOR	Inhibition	Facilitating proliferation and invasion	Advanced tumor staging	Cell culture	Wang et al. (2017)
			Inducing EMT		Clinical specimen	
miRNA-409-3p	Fip200	Inhibition	Suppressing cisplatin resistance	—	Cell culture	Cheng et al. (2018)
miR-29c-3p	FOXP1, ATG14	Inhibition	Suppressing cisplatin resistance	—	Cell culture	Hu et al. (2020)
					Animal model	
miR152	EGR1, ATG14	Inhibition	Suppressing proliferation and cisplatin resistance	Cisplatin resistance	Cell culture	He et al. (2015)
			Facilitating apoptosis		Animal model	
					Clinical specimen	
miR-1305	ARH-I, Beclin-1, VPS34	Inhibition	—	—	Cell culture	Esposito et al. (2022)
miR30a	March5, SMAD2, ATG5	Inhibition	Suppressing migration and invasion	—	Cell culture	Hu et al. (2017)
miR-30a	TGF-β, smad4, Beclin1, LC3I, LC3II	Inhibition	Facilitating cisplatin-resistant cell apoptosis	—	Cell culture	Cai et al. (2021)
miR-1251-5p	TBCC, α/β-tubulin, LC3B	Promotion	Facilitating proliferation and cycle progression	Tumor recurrence and tumor staging	Cell culture	Shao et al. (2019)
					Animal model	
					Clinical specimen	
miR-204	LC3B	Inhibition	Facilitating apoptosis	-	Cell culture	Tang et al. (2019)
			Suppressing cisplatin resistance		Animal model	
miR-20a-5p	DNMT3B, RBP1, LC3-I, LC3-II	Inhibition	Suppressing proliferation, migration and cisplatin resistance		Cell culture	Li et al. (2022)
					Animal model	
miR-4478	Fus	Inhibition	Suppressing radio-resistance		Cell culture	Wang et al. (2021b)
miR-125b	MANK2, BECLIN-1, ATG5, LC3I, LC3II	Promotion	Facilitating chemoresistance		Cell culture	Wang et al. (2021c)
					Animal model	
miR-429	ZEB1, ATG7, LC3A, LC3B	Inhibition	Facilitating apoptosis suppressing proliferation and cisplatin resistance	Overall survival (OS) and progression-free survival (PFS)	Cell culture	Zou et al. (2017)
				Cisplatin resistance	Clinical specimen	
miR-30d	ATG5, ATG12, Beclin-1, LC3B-I, LC3B-II	Inhibition	—		Cell culture	Yang et al. (2013)
miR-22	Notch1, Hes1, Beclin1, LC3 II	inhibition	Facilitating apoptosis		Cell culture	Li et al. (2018)
			Suppressing proliferation			
miR-34	Notch 1, LC3 II	promotion	Suppressing proliferation and invasion		Cell culture	Jia et al. (2019)
			Inducing apoptosis			
miR-1301	ATG5, Beclin-1	Inhibition	suppressing cisplatin resistance		Cell culture	Yu and Gao (2020)
miR-133a	YES1, LC3B	Inhibition	Suppressing cisplatin resistance		Cell culture	Zhou et al. (2022)

Numerous miRNAs can take part in the initiation stage of autophagy. The mTOR is a major player in the anabolic and catabolic processes that regulate the growth and proliferation of cells (Saxton and Sabatini, 2017). Autophagy is a catabolic process. Moreover, mTOR is an essential component of mTORC1, and mTORC1 is a negative factor of the ULK1 complex involved in initiating autophagy. The activation of mTORC1 suppresses the phosphorylation of ULK1, leading to the inhibition of autophagy (Hosokawa et al., 2009). Therefore, several miRNAs may regulate autophagy *via* the mTOR pathway. MiR8485 could bind to LAMTOR3, which resulted in decreased mTOR expression level and increased ATG13 and LC3-Ⅱ expression level, indicating the encouraging effect on autophagy (Wang et al., 2022a). MiR144-3p negatively targeted IGF2R, reduced phosphorylation levels of AKT and mTOR in OC, and promoted autophagy (Yuan et al., 2022). MiR-130a downregulated TSC1 to activate the mTOR pathway and cause the inhibition of autophagy (Wang et al., 2017). Fip200 is the first discovered interactor of the ULK complex, which is a key kinase to initiate autophagy (Hara et al., 2008). A report suggested that the Fip200-mediated autophagy could be disturbed by miRNA-409-3p (Cheng et al., 2018). ATG14 is a distinct subunit of the autophagy-associated PI3KC3 complex. It can interact with Beclin-1 *via* coiled-coil structure and enlist PI3KC3 complex into the endoplasmic reticulum, thereby promoting autophagosome formation and autophagy process (Matsunaga et al., 2010). Furthermore, it has been mentioned in the literature that ATG14 may act as a fusion medium between autophagosomes and lysosomes (Diao et al., 2015). Researchers have found that overexpressed miR-29c-3p and miR152 have direct or indirect inhibitory effects on the expression of ATG14, thus blocking autophagy progress (He et al., 2015; Hu et al., 2020). As the homologous gene of mammalian yeast ATG6, Beclin-1 is an indispensable regulator of autophagy initiation. It can induce the activation of Vps34 to form the Beclin-1-Vp34 complex, which gives an impetus to phosphatidylinositol 3-phosphate generation and lipid membrane elongation (Kihara et al., 2001). A review has suggested the role of ARH-I in triggering autophagy *via* substituting bcl-2 and binding to Beclin-1 (Lu et al., 2014). MiR-1305 could target ARH-1 to decrease the amounts of Beclin-1 and Vp34. Thus, autophagy flux was reduced by miR-1305 (Esposito et al., 2022).

There are some miRNAs participating in the expansion and completion stage of autophagy. ATG5 is an essential ingredient in forming autophagic vesicles, and its downregulation can directly lead to restricting or blocking autophagy. On the other hand, the ATG5-ATG12-ATG16 complex is involved in the regulation of autophagy as a pivotal ubiquitin-like conjugation pathway (Ye et al., 2018). TGFB1-SMAD2/3 pathway could upregulate March5 expression level *via* downregulating miR30A expression level. In addition, increased March5 levels acted as a positive regulator of ATG5 to promote autophagy (Hu et al., 2017). Another study showed that miR30A also could contribute to lower autophagy flux by inhibiting the TGF-β/Smad4 pathway (Cai et al., 2021). LC3 family, including LC3A, LC3B, and LC3C, is a human homolog of Atg8 coding gene of yeast (Wild et al., 2014). It plays a core part in a ubiquitin-like conjugation pathway of autophagy. Highly expressed miR-1251-5p might suppress LC3B expression *via* inversely modulating the expression level of TBCC and α/β-tubulin, thus impairing autophagy (Shao et al., 2019). LC3B could be inhibited by miR-204 to reduce autophagy (Tang et al., 2019). MiR-20a-5p could upregulate RBP1 expression *via* the downregulation of DNMT3B expression. Moreover, the elevated RBP1 had a remarkable restraining effect on autophagy by reducing the conversion of LC3-I to LC3-II (Li et al., 2022). Fus is a gene encoding an RNA-binding protein. Fus-containing stress particles can co-locate with the cellular autophagosomes. Accordingly, overexpression of miR-4478 could impair autophagy by suppressing fus (Wang et al., 2021b).

Furthermore, many miRNAs can act on multiple steps of autophagy to produce accumulation effects. Beclin-1, ATG5, and the conversion rate of LC3-I to LC3-II could be increased by miR125 *via* attenuating MKNK2 expression, which synergistically promoted autophagy (Wang et al., 2021c). The overexpression of miR-429 caused a decrease in anti-ATG7 and anti-LC3A/B by directly targeting ZEB1, jointly resulting in a reduction in autophagy (Zou et al., 2017). MiR-30d could inhibit multiple autophagy-associated core proteins such as Beclin-1, ATG12, ATG5, BNIP3L, and ATG2 and restrict transformation from LC3B-I to LC3B-II, leading to autophagy damage (Yang et al., 2013). Overall, different miRNAs target different autophagy-related factors to produce different effects, affecting OC progression.

### Autophagy-associated lncRNAs in OC

**FIGURE 3:**
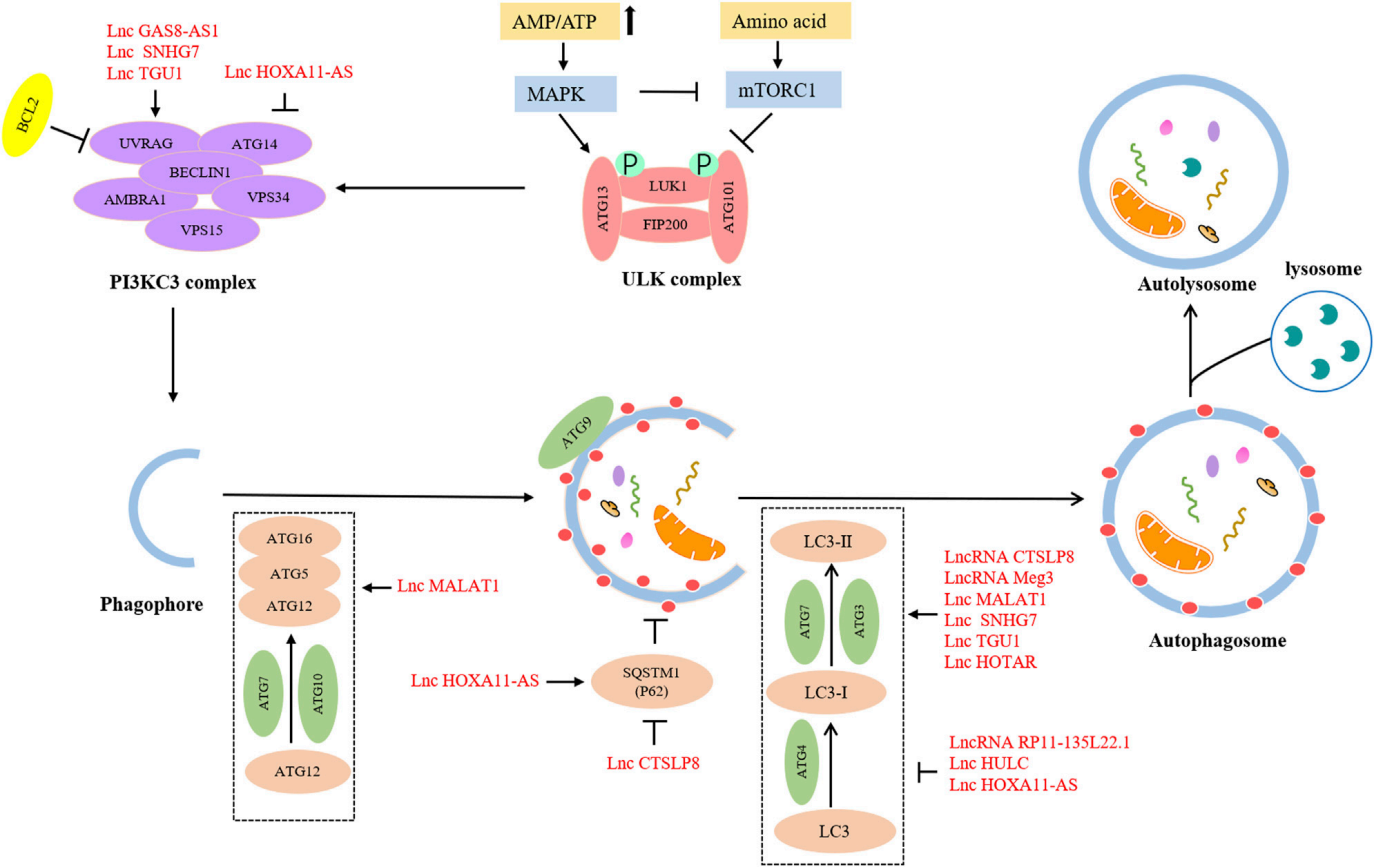
Roles of lncRNAs in autophagy regulation in OC.

The lncRNAs are aberrantly expressed in many cancers and closely related to onset, development, metastasis, and drug resistance in many cancers *via* the regulation of autophagy (Chen et al., 2017; Xin et al., 2018; Wang et al., 2019). With advances in research, some scholars have deepened their understanding of lncRNAs in the modulation of autophagy (Table 2; Figure 3).

**TABLE 2:** LncRNAs involved in autophagy modulation in OC.

LncRNA	Possible targets	Autophagic effects	Effects on OC cells	Effects on OC samples	Models	Reference
CTSLP8	CTSLP8, miR-199a-5p, CTSL1, SQSTM1 (p62) LC3	Promotion	Facilitating proliferation, migration, and invasion inducing EMT	Tumor staging and tumor grading	Cell culture	Wang et al. (2021a)
					Animal model	
					Clinical specimen	
HULC	ATG7, LC3, SQSTM1 (P62), LAMP1	Inhibition	Facilitating proliferation, migration and invasion	—	Cell culture	Chen et al. (2017)
			Suppressing apoptosis		Animal model	
					Clinical specimen	
GAS8-AS1	Beclin1	Promotion	Suppressing growth, migration and invasion	Five-year survival rate	Cell culture	Fang et al. (2020)
					Clinical specimen	
Meg3	ATG3	Promotion	Suppressing and colony formation	Tumor staging	Cell culture	Xiu et al. (2017)
			Inducing cell cycle arrest in G2 phases		Clinical specimen	
			Promoting apoptosis			
SNHG7	miR-3127-5p, LC3-II, Beclin1	Promotion	Suppressing apoptosis	—	Cell culture	Yu et al. (2022)
			Facilitating migration and invasion			
TUG1	miR-29b-3p, Beclin-1, LC3B-I, LC3B-II	Promotion	Facilitating chemoresistance	Tumor staging	Cell culture	Gu et al. (2020)
				Drug resistance	Clinical specimen	
HOXA11-AS	LC3B-I, LC3B-II, Beclin1, P62	Inhibition	Facilitating proliferation, migration, invasion and cisplatin resistance suppressing apoptosis	—	Cell culture	Chen et al. (2022b)
HOTAIR	ATG7, LC3B-I, LC3B-II	Promotion	Facilitating cisplatin resistance	—	Cell culture	Yu et al. (2018b)
MALAT1	TGFβR2, Smad2/3, March7, ATG7	Promotion	Facilitating migration and invasion	—	Cell culture	Hu et al. (2018)
					Animal model	
					Clinical specimen	
RP11-135L22.1	RP11-135L22.1, ATG7, LC3-I, LC3-II	Inhibition	suppressing tumor proliferation facilitating apoptosis		Cell culture	Zou et al. (2018)

In the initiation stage, overexpressed lncRNA GAS8-AS1 interacted with Beclin-1 to trigger autophagy (Fang et al., 2020). In the expansive and completion stage, lncRNA Meg3 might upregulate the level of ATG3 by forming Meg3-ATG3 complex and trigger ATG3-dependent autophagy (Xiu et al., 2017). Moreover, more lncRNAs can affect multiple procedures of autophagy. The lncRNA SNHG7 served as a sponge of miR-3127-5p to active autophagy *via* upregulating Beclin-1 and LC3-II expression levels (Yu et al., 2022). LncRNA TUG1 could directly bind to miR-29b-3p and increase the expression of Beclin-1 and convert LC3B-I to LC3B-II to promote autophagy (Gu et al., 2020). In addition to declining the expression of Beclin-1 and LC3-II, lncRNA HOXA11-AS also could increase the p62 expression level to suppress the autophagy process (Chen et al., 2022b). ATG7 expression could be upregulated to facilitate autophagy by lncRNA HOTAIR and lncRNA MALAT1. However, lncRNA HOTAIR could result in an increased ratio of LC3-II to LC3-I (Yu et al., 2018b; Hu et al., 2018). Although lncRNA HULC and lncRNA RP11-135L22.1 could downregulate ATG7 and LC3 -II expression to impair autophagy, lncRNA HULC-mediated ATG7 downregulation also could induce the expression of LAMP1 to suppress autophagy (Chen et al., 2017; Zou et al., 2018). Collectively, these studies present strong evidence that lncRNAs-associated signaling pathways have impacts on OC development by regulating cell autophagy.

### Autophagy-associated circRNAs in OC

It is more than 40 years since the first discovery of circRNA in plant viroids (Sanger et al., 1976). After then, accumulating data indicate that circRNAs perform vital roles in the initiation and progression of OC through modulating autophagy. For example, the direct binding of circMUC16 and miR-199a-5p alleviated the expression inhibition of RUNX1, which, in turn, caused increased circMUC16 expression. Additionally, circMUC16 could combine with ATG13 and facilitate its expression, and overexpressed circMUC16 could promote autophagy (Gan et al., 2020). The interaction between circEEF2 with miR-6881-3p and ANXA2 has been implicated in autophagy. The former interaction upregulated the ATG5 and ATG7 expression, and the latter downregulated the expression of p-mTOR, resulting in the promotion of autophagy (Yong et al., 2020). Furthermore, circRAB11FIP1 regulated ATG14 and ATG7 by sponging miR-129 or binding with DSC1 protein to promote autophagy (Zhang et al., 2021). Taken together, circRNAs function as a sponge to interact with miRNAs, regulating the autophagy of OC.

### Prognosis values of autophagy-associated ncRNAs in OC patients

A growing body of evidence has shown that several autophagy-associated ncRNAs may be crucial players in the prognosis of OC patients, and they have great potential to become prognostic biomarkers and treatment targets. Recently, based on abnormally expressed autophagy-related lncRNAs, Li et al. have proposed an autophagy-associated lncRNA risk model for the prediction of prognosis and therapeutic effect in OC patients (Li et al., 2021). However, there is no substantive data to prove that they are involved in the specific roles of autophagy regulation in the occurrence and development of ovarian cancer.

Up to now, only a small number of ncRNAs have been detailly revealed in the OC outcome. For instance, lncRNA GAS8-AS1 interacted with Beclin-1 to promote autophagy, and its deletion caused an enhanced ability of OC cells to metastasize, invade, and proliferate (Fang et al., 2020). An elevation of lncRNA RP11-135L22.1 showed a prohibitive role in cisplatin-induced autophagy, leading to the reduction of cell proliferation and increased cell apoptosis (Zou et al., 2018). Similarly, high expression of miR-22 and miR-34 could suppress cell proliferation and expedite cell apoptosis *via* the downregulation of the notch signaling pathway (Li et al., 2018; Jia et al., 2019). The suppressive effects of radiation on the growth of OC cells could be reinforced, and the response of DNA damage repair to radiation could be attenuated, thus improving the sensitivity of OC cells to radiotherapy by upregulating miR-4478 to downregulate Fus and reduce autophagy (Wang et al., 2021b). NF-κB signaling pathway could be inhibited by miR-1301 to attenuate cisplatin resistance and delay the development of EMT in OC, ultimately refraining the progression of OC (Yu and Gao, 2020). Additionally, autophagy could be suppressed by miR-133a and miR-1301, thereby suppressing cisplatin resistance (Yu and Gao, 2020; Zhou et al., 2022). More importantly, the imbalance of ncRNA homeostasis was also closely related to the clinical characteristics of OC patients. For example, miR-130a was upregulated and could promote OC progression through inhibiting TSC3 expression in OC samples. The aberrant miR-130a-TSC3 axis has also been proved to be associated with the patients’ tumor stage (Wang et al., 2017). He and colleague reported an autophagy-associated miRNA, miR-152, in cisplatin-resistant OC samples. This group found that of miR-152 overexpression could weaken cisplatin resistant by preventing cyto-protective autophagy and enhancing cell apoptosis (He et al., 2015). In addition, the expression of LncMeg3 was significantly downregulated in OC specimens, and was negatively correlated with stage of OC patients (Xiu et al., 2017). Based on these findings, clarifying the detailed roles of autophagy-associated ncRNAs may become a new direction for clinical management of OC patients.

### Involvement of ncRNA-autophagy axis in immune regulation

Previous studies have suggested the involvement of autophagy in immune regulation (Jiang et al., 2019; Wang et al., 2022b; Xie et al., 2022). The autophagic decomposition of cyclic GMP-AMP synthase could be astricted by TRIM14, thus enhancing congenital immunity (Chen et al., 2016). Autophagy blocking has an intensive effect on programmed death ligand-1 (PD-L1)-associated immunological suppression in bladder cancer (Tsai et al., 2022). With progress in research, the findings have indicated that ncRNA-mediated autophagy is involved in immunomodulation (Eng et al., 2021). Mir223 could suppress autophagy flux by lowering ATG16L1 expression, causing aggravation of inflammation in the central nervous system (Li et al., 2019). The tumor-infiltrating lymphocyte of CD4 was expressed highly under the condition of autophagy suppression by modulating miR-155 and activating TNF‐related apoptosis‐inducing ligand in lung cancer (Zarogoulidis et al., 2016). Several autophagy-associated lncRNAs are related to the immune microenvironment. They may be candidates for predicting the prognosis of OC patients (Zhang et al., 2022b). Collectively, the double roles of ncRNAs in autophagy and immunomodulation determine their importance in cancers, providing a theoretical basis for future cancer immunotherapy.

## Conclusion and perspectives

The ncRNAs participate in the oncogenesis and progression of cancers through regulating cell autophagy, although the specific mechanisms still need to be further explored. These autophagy-associated ncRNA might be conductive to suppress or facilitate cell growth, and affect the therapeutic response. Therefore, it is reasonable to speculate that ncRNA inhibitors or activators can be developed to improve therapeutic effect and prolong the survival of OC patients. In addition, some ncRNAs could be stably expressed in the biological fluids of tumors, and participate in the regulation of cell autophagy (Keller et al., 2011). Serving as the independent indicators of prognosis, these ncRNAs in liquid biopsy can predict the recurrence and disease-free survival rates of cancer patients. Thus, the strategies targeting autophagy-associated ncRNAs would provide novel therapeutic concepts and application prospects for OV cancers.
